# Evolution of Spanish population well-being during the COVID-19 pandemic: Results from the COSMO-Spain study

**DOI:** 10.1016/j.heliyon.2025.e42409

**Published:** 2025-01-31

**Authors:** Catarina Santos-Ribeiro, Carmen Rodríguez-Blázquez, Alba Ayala, María Romay-Barja, María Falcón, Maria João Forjaz

**Affiliations:** a Preventive Medicine Department, Hospital Universitario 12 de Octubre, Madrid, Spain; b Escuela de Doctorado, Universidad Autónoma de Madrid, Spain; c National Epidemiology Centre, Carlos III Health Institute, Madrid, Spain; d Network Centre for Biomedical Research in Neurodegenerative Diseases (CIBERNED), Madrid, Spain; e Nursing and Health Care Research Unit (Investén‐isciii), Carlos III Health Institute, Spain; f Research Network on Chronicity, Primary Care, prevention and health Promotion (RICAPPS), Madrid, Spain; g National Centre of Tropical Medicine, Carlos III Health Institute, Madrid, Spain; h Legal Medicine, Department of Sociosanitary Sciences, University of Murcia, Murcia, Spain

**Keywords:** WHO-5, Well-being, COVID-19, Coronavirus, Pandemic

## Abstract

**Introduction:**

The COVID-19 pandemic affected mental health worldwide. The COSMO-Spain study analyses risk perceptions, behaviours, knowledge and other pandemic related variables, such as well-being. This work aimed to assess the evolution of self-reported well-being in Spain from May 2021 to September 2022 and its association with demographic and COVID-19 related factors.

**Methods:**

An online, nationwide cross-sectional panel survey was applied in seven rounds with 1000+ participants each, with a total sample of 7266 participants, representative of the Spanish adult general population. The main variable was well-being, measured with the World Health Organization Well-Being Index (WHO-5) total score, an index with a total score from 0 to 100 (0 = worst well-being, 100 = best well-being). Other variables included in the survey were: sociodemographic data, concern about COVID-19, feelings of depression and fear, COVID-19-related worries, risk perception, self-efficacy, preventive behaviours, pandemic fatigue, health literacy, information search behaviours, and trust in several institutions. A multiple linear regression was run to analyse the associated factors with the WHO-5 total score.

**Results:**

The WHO-5 total score showed a significant increase from rounds 6 (May–June 2021) to 8 (September–October 2021). Women (standardized b coefficient (b) = -0.10), youth or people with lower socioeconomic status (worsened *financial situation* (b = −0.10) or unemployed/furloughs (b = −0.04)) reported lower well-being levels, whereas having a university-level education showed the opposite (b = 0.11). Feeling less depressed was associated with higher well-being (b = 0.31).

**Conclusions:**

This study shows rising levels of well-being until a plateau was reached in October 2021. Vulnerable groups may be at higher risk of worsened mental health and should be addressed by policymakers. Further longitudinal studies should evaluate causality and evolution patterns of well-being throughout the COVID-19 pandemic.

## Highlights

• Well-being increased in the 2nd year of the pandemic but remained lower than before.
• Vulnerable groups were youth, women and people with lower socioeconomic status.
• Self-reported feelings of depression and well-being seem to have a key association.
• Increases in well-being may be promoted by empowering and educating people.
• Well-being is a complex, subjective construct, influenced by a multitude of KAPs.

## 1 Introduction

On December 2019, the novel Severe Acute Respiratory Syndrome Coronavirus 2 (SARS-CoV-2) was detected in China [1]. Three months later, the World Health Organization (WHO) declared Coronavirus Disease 2019 (COVID-19) as a global pandemic and Spain decreed a firm population-wide lockdown, which lasted until June 2020 [2]. This was followed by a period of restrictive and preventive measures established locally, according to the epidemiological situation [3]. The pandemic economic impact during the first year was profound [4], with increasing rates of unemployment and furloughs (in Spanish “expedientes de regulación temporal de empleo” or ERTE). New waves of coronavirus infections led to a temporary reinforcement of mitigation measures from October 2020 [3] to May 2021.

From the moment the first lockdown was enforced, WHO's strategy to control SARS-CoV-2 spread focused on three non-pharmacological interventions (NPI), the 3 W's [5]: washing hands, wearing a face mask and watching social distance. Meanwhile, pharmacological interventions were developed and vaccination in Spain started on December 27th^,^ 2020 [6], firstly targeting at-risk groups and gradually extending to the general population, with vaccination acceptance increasing from 43.1 % in September 2020 to 84.5 % in May 2021 [7]. By September 2021, 70 % of the population was fully vaccinated [8] and booster doses had started being administered. One year later, 85.6 % of the Spanish population had completed the initial vaccination protocol. Mortality rates during the three pandemic waves in 2021 were significantly lower than the preceding year [8].

Pandemics, along with the restrictions and NPIs implemented to curb its spread, are known to affect population health [9], compromising the “state of complete physical, mental and social well-being" [10]. Even though there is no consensual definition [11], well-being is a complex multidimensional construct that may reflect mental health in a broader sense, evaluating people's perceptions of being cheerful, calm, active, fresh, day filled with interesting things [12]. It can be measured using different scales such as Warwick-Edinburgh Mental Wellbeing Scale [13], the Psychological General Well-Being Index [14] or the WHO Well-being Index (WHO-5), a questionnaire used to assess subjective self-reported positive well-being in 5 items, validated in multiple languages and widely used [15].

During the first year of this pandemic, several studies analysed the impact of behavioural recommendations and restrictive measures enforced by governments [12,16,17] on mental health in this broader context. Overall, they found well-being levels were lower than pre-pandemic reference values [[16], [17], [18]], with a significant decrease in well-being observed in Spain, and WHO-5 total scores barely surpassing 50 out of 100 [18]. Furthermore, being young, a woman, a student or having low income [12,16,17] during the lockdown impacted negatively on well-being, while being employed had the opposite effect [16,17,19]. Depression and anxiety have risen worldwide since 2020 [[20], [21], [22]], showing a negative correlation with well-being as well [[21], [22], [23]]. COVID-19-related aspects such as pandemic fatigue, the feeling of distress that may lead to a lack of motivation to follow recommended protective measures, seem to follow the same trend [24]. Furthermore, the perceived risk of contagion and severity, if infected, have shown negative associations with well-being, whereas perceived self-efficacy to avoid infection associates with better mental health [12,25].

Other factors with a negative association with well-being are digital media use [26,27], high frequencies of COVID-19 information search [27] and high levels of misinformation [28]. Increased knowledge on COVID-19 health-related topics has been associated with higher well-being levels [1,27], as do trusting in information sources, institutions and organizations [12].

The WHO Regional Office for Europe launched the COVID-19 Snapshot MOnitoring (COSMO) study [12] to evaluate the population's knowledge, attitudes, preventive practices (KAP) and well-being during the pandemic. Spain joined this study in May 2020, with COSMO-Spain [25] surveys results being publicly available [3].

Even though evidence regarding psychological well-being during the first year of the pandemic is abundant [20,29], data regarding the second year of the pandemic are scarce. The present work aims to assess the evolution of self-reported psychological well-being by the Spanish population from May 2021 to September 2022, and its association with demographic characteristics and COVID-19-related factors.

## 2 Materials and methods

### 2.1 Study protocol

The COSMO-Spain study consists of a nationwide, cross-sectional panel survey on the KAP, risk perception and psychological variables related to the COVID-19 pandemic [25,30]. It has been conducted approximately every 2 months by a consumer research company, which recruits a sample representative of the Spanish population regarding age, gender, education level and large area of residence, in order to address potential selection biases. The quotas that were used to define representative groups were based on the Spanish population distribution reported by the Spanish National Statistics Institute [31]. An email invitation to answer an online survey was sent to the members of the company's panel aged 18 or older living in Spain. People who did not respond were replaced by others in the same group. Quality control measures implemented by the consumer research company include soft launches for each round and control questions to detect inconsistencies. Overall, each round counted with 1.000+ participants, as defined by the WHO protocol [12], and statistical weighting was used to correct some quotas that were not completely fulfilled during the recruitment period for each round (Table 1). The current work analysed data rounds 6 to 12, as rounds 1 to 5 were conducted during the first year of the SARS-CoV-2 pandemic. Table 1 summarizes each round timeline, as well as data regarding the epidemiological context at each moment [[32], [33], [34], [35], [36], [37], [38]] and response rates.

**Table 1:** COSMO-Spain rounds 6 to 12 characterization.

	Round 6	Round 7	Round 8	Round 9	Round 10	Round 11	Round 12
Dates	May 24th to June 3rd^,^ 2021	July 26th to August 5th^,^ 2021	September 27th to October 4th^,^ 2021	November 30th to December 13th^,^ 2021	February 25th to March 4th^,^ 2022	April 29th to May 4th^,^ 2022	September 21st to 29th 2022
*Number of cases in the previous fortnight*	94,236 [34]	291,765 [35]	25,682 [36]	153,298 [37]	290,544 [38]	83,323 [39] a	18,458 [40] a
14-day accumulated incidence	198.60/100,000 inhabitants [34]	614.88/100,000 inhabitants [35]	54.12/100,000 inhabitants [36]	323.07/100,000 inhabitants [37]	613.15/100,000 inhabitants [38]	676.43/100,000 inhabitants [39] a	149.84/100,000 inhabitants [40] a
*Invitations*	3364	3701	5334	2894	1302	1308	1163
Answers	1727	1334	2557	1794	1118	1204	1163
*Complete Surveys (n)*	1001	1000	1042	1049	1067	1056	1051
Statistical weight	97.33 %	99.87 %	96.85 %	98.42 %	96.80 %	95.50 %	98.07 %
*Sampling error*	3.14 %	3.10 %	3.08 %	3.05 %	3.02 %	3.02 %	3.01 %

a Data limited to people over 60 years old.

As a whole, a total of 19,066 invitations to participate were sent via electronic mail, reaching a total of 7266 completed surveys. For each round, a new sample of participants was drawn from a pre-existing panel, ensuring that each person was only able to answer the survey once, thus guaranteeing independence of observations.

### Ethical statement

This study was approved by the Carlos III Health Institute Ethics Committee (CEI PI 59_2020-v2), dated July 14th^,^ 2020. Participants were informed about the study goals and consented both to fulfilling the questionnaire and to the publication of the anonymized aggregated results [3] by checking a box.

### 2.2 Measures

#### 2.2.1 Sociodemographics

Demographic variables, such as *sex*, *age group*, *education level*, *economic situation* during the previous three months and *employment status* were collected as shown in Supplementary Table 1. *Employment status* results from merging two variables from the original COSMO-Spain survey [30]: *employment* (“working”, “student”, “homemaker”, “retired/pensioner”, “long-term unemployed”, “unemployed or ERTE”) and *type of work (“with high risk of contagion”, “*with moderate risk of contagion”, “no risk”, “telework” and “healthcare staff”). The resulting variable has the following categories: working a high to moderate risk of contagion job; working a low risk of contagion job; student; retired/pensioner/homemaker; unemployed/ERTE.

#### 2.2.2 WHO-5

The WHO-5 questionnaire is an adaptation of the WHO-10 well-being index containing only positively phrased items [39], that has been translated and validated into more than 30 languages, including Spanish [40], with satisfactory psychometric properties and a high content validity [15,40]. This questionnaire requires participants to recall if in the two weeks prior to answering they felt (a) cheerful and in good spirits, (b) calm and relaxed, (c) active and vigorous, (d) woken up feeling fresh and rested and (e) a daily life filled with things that interest them. Each item is scored on a scale from 0 (at no time) to 5 (all of the time). The raw score ranges from 0 to 25 and can be multiplied by 4, resulting in a total score where 0 is the worst possible well-being and 100 the best. Cut-off scores under 50 out of 100 can be suggestive of clinical depression [40].

#### 2.2.3 Worries

There were several items used to evaluate people's worries and concerns regarding the COVID-19 pandemic. *Concern about COVID-19* was evaluated by asking the participants “how concerned are you about coronavirus/COVID-19”. Answering possibilities ranged from 1 (not concerned at all) to 5 (extremely concerned). Perceived *speed of propagation* was scored from 1 (spreading slowly) to 5 (spreading fast). *Feelings of depression* and feelings of *fear* were also enquired with two items scored from 1 (“makes me feel depressed” or “fear”, respectively) to 5 (“does not affect my mood”, in case of depression, and “does not make me feel fear at all”, in case of fear). Worries about specific situations were scored using a scale from 1 (not worried at all) to 5 (worried a lot). The included situations were: a *health system overload*, their *own physical and mental health*, *going outside*, *a new lockdown*, *losing a loved one*, *becoming unemployed* and *new coronavirus variants*.

#### 2.2.4 Risk perception

*Risk perception* was evaluated with three different items, all of which were answered in 5-point scales: *severity*; *probability* and *self-efficacy at avoiding COVID-19*. Perceived severity in case of COVID-19 contagion was rated from 1 (very light) to 5 (very severe), as was the probability of coronavirus contagion (from highly unlikely to highly likely). Finally, self-efficacy was assessed with the question “nowadays, avoiding coronavirus/COVID-19 infection, for me is …” and possible answers ranged from 1 (very hard) to 5 (very easy).

#### 2.2.5 Preventive behaviours

Four items were used to evaluate compliance with preventive behaviours, each one was scored from 1 (never) to 5 (always). Participants were asked how often in the previous 7 days they washed their hands with soap and water or with hydroalcoholic gel, how often they kept a physical distance of at least 2 m and how often they used a face mask correctly.

In the final round, face mask correct use is a result of the mean value of two different items: “using face mask following recommendations in health centres, nursing homes and pharmacies” and “in public transport”, which were asked separately.

#### 2.2.6 Pandemic fatigue

*Pandemic fatigue* was assessed using the COVID-19 Pandemic Fatigue Scale (CPFS), a tool recently validated for the Spanish population [41], composed of six items: “I am tired of all the COVID-19 discussions in TV shows, newspapers and radio programs, etc.”, “I feel strained from following all of the behavioural regulations and recommendations around COVID-19”, “I am sick of hearing about COVID-19”, “I am tired of restraining myself to save those who are most vulnerable to COVID-19”, “when friends or family members talk about COVID-19, I try to change the subject because I do not want to talk about it anymore” and “I am losing my spirit to fight against COVID-19”. Each item was scored from 1 (strongly disagree) to 5 (strongly agree). The CPFS total sum score ranged from 6 to 30, where higher scores represented higher levels of pandemic fatigue.

#### 2.2.7 Health literacy

*Health literacy* was assessed using 7 items asking participants how easy it was for them to: *follow recommendations about protecting oneself, value if media information is reliable, understand what to do when one is a close contact, find information regarding vaccines, understand the risks and benefits of vaccination, assess if media information regarding vaccines is reliable and decide if one should get vaccinated*. The response scale was from 1 – very difficult to 4 – very easy. These items were selected from the validated COVID-19 Health Literacy Questionnaire [42].

#### 2.2.8 Information seeking behaviour and trust in information sources

Respondents were asked how often they searched for information regarding coronavirus/COVID-19 (*information search frequency*) ranking from 1 (never) to 5 (several times a day). Participants were then inquired about their trust in the information provided by different sources: *TV news*, *debate programs*, *press conferences, national press, healthcare professionals, social media,* the *internet,* the *Health Ministry,* the *WHO,* COVID-19 *help phone lines* and the *radio.* The level of trust ranged between 1 (very little trust) and 5 (the highest level of trust).

#### 2.2.9 Trust in institutions

Participants were asked to rate their trust in several institutions and organizations. Possible responses were scored on a five-point scale where the lowest value [1] meant no trust and the highest value [5] represented a strong sense of trust. The evaluated institutions were: *primary care centres, the workplace, hospitals,* the *Ministry of Health,* the *Regional Government, scientists, education centres, public transport, press* and the *Central Government.*

All variables are summarized in Supplementary Table 1.

### 2.3 DATA analysis

Descriptive statistics were applied to every variable in the study, both overall and by round. For binary and categorical variables, the percentage of participants that selected each option was computed. Mean and standard deviation (SD) were calculated for continuous variables. Ordinal variables, evaluated using response scales, were analysed as continuous.

Visual inspection of the histogram of the main variable (WHO-5) revealed an approximation to normality. A one-way ANOVA with a Bonferroni post hoc test was conducted to identify differences in WHO-5 total score by rounds.

A multiple linear regression was run using WHO-5 total score as the dependent variable, with a total sample of 6203 respondents. Independent variables used were the ones described in Supplementary Table 1, with the exception of *trust in sources of information* and *trust in institutions*, due to the very strong Pearson correlation (r) several of its items showed amongst each other (r > 0.60). A total of 1063 surveys from the 7266 answered questionnaires were excluded due to missing values when answering one or more Health Literacy items. Independence of observations was assured during the study design and multicollinearity statistics were assessed and its absence guaranteed. Homoscedasticity and normality assumptions were met. Data analysis was performed using IBM SPSS Statistics, Version 28.0 and R, Version 4.4.1.

## 3 Results

### 3.1 Sociodemographic profile

Table 2 features the total descriptive statistics for every variable and Supplementary Table 2 depicts descriptive statistics by round. Almost two-thirds of the participants were 35–64 years old (64.7 %, n = 4647). Most of the participants either had primary (27.2 %; n = 1976) or university-level education (32.0 %; n = 2329). Over two-thirds of the participants felt their economic situation during the previous three months remained the same (64.9 %, n = 4713). Regarding employment status, 38.0 % of the respondents reported working in high-to-moderate risk of contagion jobs, while 14.1 % were unemployed/ERTE.

**Table 2:** Descriptive statistics of the study variables.

***Variable***	Categories	**Total (n=7266)**	
		**n**	**%**
***Sex***	Female	3633	50.0 %
	Male	3633	50.0 %
***Age group****(years old)*	18 to 24	497	6.8 %
	25 to 34	1251	17.2 %
	35 to 49	2403	33.1 %
	50 to 64	2244	30.9 %
	65+	871	12.0 %
***Education level***	Incomplete primary or less	1239	17.1 %
	Primary	1976	27.2 %
	Secondary	1722	23.7 %
	University	2329	32.0 %
***Economic situation*** (during the previous three months)	Has improved	700	9.6 %
	Remains the same	4713	64.9 %
	Has worsened	1854	25.5 %
***Employment status***	Working a high to moderate risk of contagion job	2758	38.0 %
	Working a low risk of contagion job	1190	16.4 %
	Student	474	6.5 %
	Retired/pensioner/homemaker	1821	25.0 %
	Unemployed/ERTE	1024	14.1 %
		**Mean**	**Standard deviation**

***WHO-5 total score*** (0–100)		54.49	22.81
***Concern about COVID-19*** [[1], [2], [3], [4], [5]]		3.10	1.15
***Speed of propagation*** [[1], [2], [3], [4], [5]]		3.44	1.21
***Feelings of depression*** [[1], [2], [3], [4], [5]]		3.07	1.25
***Feelings of fear*** [[1], [2], [3], [4], [5]]		3.01	1.24
**Worry** [[1], [2], [3], [4], [5]]	*Health system overload*	4.19	1.07
	*Own physical and mental health*	3.73	1.30
	*Going outside*	2.55	1.32
	*A new lockdown*	3.68	1.39
	*Losing a loved one*	4.44	1.01
	*Becoming unemployed*	3.07	1.63
	*New coronavirus variants*	3.86	1.19
***Risk perception: severity*** [[1], [2], [3], [4], [5]]		2.87	0.96
***Risk perception: probability*** [[1], [2], [3], [4], [5]]		2.84	1.12
***Self-efficacy at avoiding COVID-19*** [[1], [2], [3], [4], [5]]		3.18	1.00
***Frequent hand hygiene*** [[1], [2], [3], [4], [5]]		3.99	1.17
***Hydroalcoholic gel use*** [[1], [2], [3], [4], [5]]		3.99	1.23
***Physical distancing*** [[1], [2], [3], [4], [5]]		3.66	1.21
***Using masks*** [[1], [2], [3], [4], [5]]		4.51	0.92
***Pandemic fatigue*** [[6], [7], [8], [9], [10], [11], [12], [13], [14], [15], [16], [17], [18], [19], [20], [21], [22], [23], [24], [25], [26], [27], [28], [29], [30]]		17.71	5.44
**Health literacy** [[1], [2], [3], [4]]	*Following recommendations about protecting oneself*	3.29	0.71
		(n = 7089)	
	*Valuing if media information is reliable*	2.58	0.94
		(n = 6772)	
	*Understanding what to do when one is a close contact*	3.17	0.76
		(n = 7037)	
	*Finding information regarding vaccines*	2.96	0.87
		(n = 6896)	
	*Understanding risks and benefits of vaccination*	3.20	0.85
		(n = 6979)	
	*Assessing if media information regarding vaccines is reliable*	2.62	0.95
		(n = 6755)	
	*Deciding if one should get vaccinated*	3.38	0.84
		(n = 7015)	
***Information search frequency*** [[1], [2], [3], [4], [5]]		2.72	1.08
**Trust in sources of information** [[1], [2], [3], [4], [5]]	***TV News***	2.76	1.17
	***Debate programs***	2.50	1.12
	***Press conferences***	2.67	1.15
	***National press***	2.67	1.10
	***Healthcare professionals***	3.87	1.11
	***Social media***	2.05	1.06
	***Internet***	2.42	1.06
	***Health Ministry***	3.22	1.27
	***WHO***	3.25	1.24
	***Help phone lines***	3.07	1.20
	***Radio***	2.78	1.10
**Trust in institutions** [[1], [2], [3], [4], [5]]	***Primary care centres***	3.43	1.13
	***Workplace***	3.06	1.15
	***Hospitals***	3.60	1.11
	***Health Ministry***	3.06	1.26
	***Regional Government***	2.98	1.16
	***Scientists***	3.83	1.12
	***Education centres***	3.10	1.06
	***Public transport***	2.39	1.10
	***Press***	2.44	1.06
	***Central Government***	2.54	1.25

### 3.2 WELL-BEING throughout the rounds

**Fig. 1:**
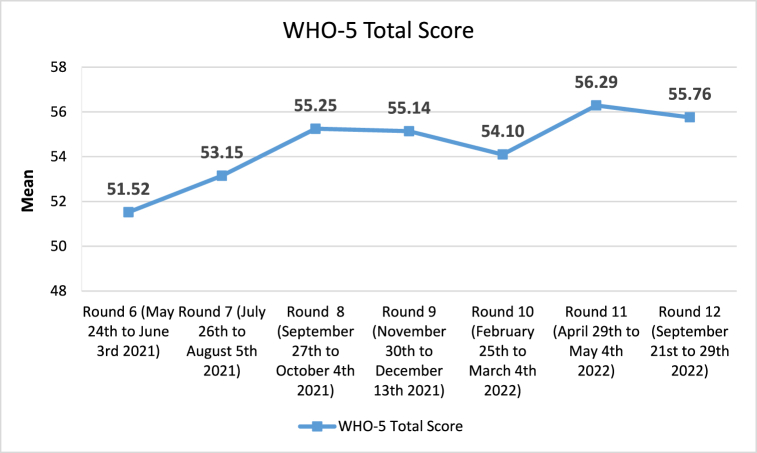
WHO-5 total score evolution by round.

Fig. 1 portrays the evolution of the average WHO-5 total score throughout all seven rounds. There was a significant linear trend during the seven rounds (p < 0.001), and statistically significant increases in WHO-5 total score from round 6 to rounds 8 (mean difference (MD) = 3.73, p = 0.004), 9 (MD = 3.62, p = 0.007), 11 (MD = 4.78, p < 0.001) and 12 (MD = 4.24, p = 0.001). There were no significant differences between round 8 and round 12.

### 3.3 Factors associated with well-being

**Fig. 2:**
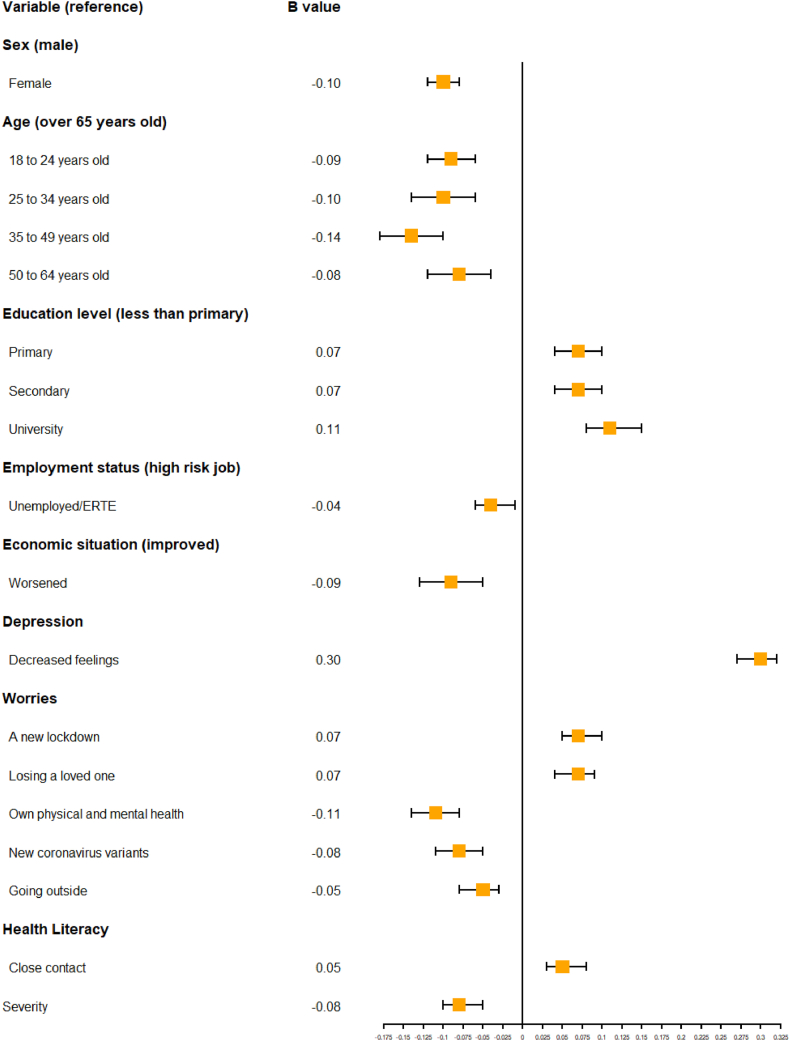
Standardized beta (b) values for sociodemographic variables and other variables significantly associated with well-being.

Regarding sociodemographic variables, increases in education level showed the highest positive association with the *WHO-5 total score*, especially in case of university-level education (standardized beta coefficient (b) = 0.11), when compared with incomplete primary education. Sex, age and employment status, however, had the opposite effect. Being younger showed a negative association with the *WHO-5 total score* in all age groups when compared with being over 65 years old, particularly for 35 to 49-year-olds (b = −0.14). Females also reported lower *WHO-5 total scores* (b = −0.10, p < 0.001), as did people with worsened *financial situation* (b = −0.10). The regression model of WHO-5, with an explained variance of 27.2 %, can be found in Table 3. Fig. 2 shows sociodemographic variables and other variables (the ones with higher or lower b values) significantly associated with well-being.

**Table 3:** Multiple linear regression results with unstandardized and standardized coefficients.

	Unstandardized Coefficients		Standardized Coefficients	t	Sig.
	B	Std. Error	b		
(Constant)	18.72	3.26		5.75	<0.001
*Round 7*	1.88	0.95	0.03	1.97	0.049
*Round 8*	2.58	0.93	0.04	2.77	0.006
*Round 9*	3.16	0.94	0.05	3.37	0.001
*Round 10*	1.42	0.95	0.02	1.49	0.136
*Round 11*	2.39	0.97	0.04	2.45	0.014
*Round 12*	0.75	1.01	0.01	0.74	0.457
*Sex*	−4.41	0.53	−0.10	−8.39	<0.001
*18 to 24 years old*	−8.33	1.58	−0.09	−5.27	<0.001
*25 to 34 years old*	−6.07	1.16	−0.10	−5.21	<0.001
*35 to 49 years old*	−6.78	1.024	−0.14	−6.62	<0.001
*50 to 64 years old*	−3.80	0.94	−0.08	−4.04	<0.001
*Education level = Primary*	3.41	0.78	0.07	4.38	<0.001
*Education level = Secondary*	3.74	0.80	0.07	4.65	<0.001
*Education level = University*	5.39	0.81	0.11	6.69	<0.001
*Employment status = Working a low risk of contagion job*	0.11	0.73	0.002	0.15	0.880
*Employment status = Student*	−1.98	1.33	−0.02	−1.49	0.137
*Employment status = Retired/pensioner/homemaker*	−0.74	0.79	−0.01	−0.94	0.345
*Employment status = Unemployed/ERTE*	−2.36	0.80	−0.04	−2.93	0.003
*Economic situation =* Remains the same	−0.77	0.87	−0.02	−0.89	0.375
*Economic situation =* Has worsened	−4.64	0.98	−0.09	−4.74	<0.001
*Concern*	−0.28	0.29	−0.01	−0.96	0.338
*Speed of propagation*	0.15	0.24	0.01	0.64	0.522
*Depression*	5.38	0.25	0.30	21.79	<0.001
*Fear*	0.27	0.26	0.01	1.05	0.292
*Health system overload*	0.75	0.31	0.035	2.45	0.014
*Own physical and mental health*	−1.89	0.25	−0.11	−7.69	<0.001
*Going outside*	−0.94	0.23	−0.05	−4.09	<0.001
*A new lockdown*	1.19	0.20	0.07	5.82	<0.001
*Losing a loved one*	1.49	0.32	0.07	4.70	<0.001
*Becoming unemployed*	0.13	0.18	0.01	0.73	0.463
*New coronavirus variants*	−1.58	0.31	−0.08	−5.16	<0.001
*Severity*	−1.80	0.30	−0.08	−6.04	<0.001
*Likelihood of contagion*	0.04	0.24	0.002	0.16	0.870
*Self-efficacy*	0.69	0.25	0.03	2.76	0.006
*Frequent hand hygiene*	0.78	0.28	0.04	2.80	0.005
*Hydroalcoholic gel usage*	0.41	0.27	0.02	1.49	0.137
*Physical distancing*	0.62	0.27	0.03	2.26	0.024
*Using masks*	−0.29	0.35	−0.01	−0.82	0.412
*Pandemic fatigue*	−0.07	0.05	−0.02	−1.43	0.154
*Health literacy = Following recommendations about protecting oneself*	1.53	0.43	0.05	3.54	<0.001
*Health literacy = Valuing if media information is reliable*	0.41	0.39	0.02	1.04	0.300
*Health literacy = Understanding what to do when one is a close contact*	1.61	0.39	0.05	4.15	<0.001
*Health literacy = Finding information regarding vaccines*	0.47	0.39	0.02	1.22	0.223
*Health literacy = Understanding risks and benefits of vaccination*	−0.64	0.41	−0.02	−1.58	0.115
*Health literacy = Assessing if media information regarding vaccines is reliable*	0.42	0.40	0.02	1.04	0.298
*Health literacy = Deciding if one should get vaccinated*	0.50	0.38	0.02	1.31	0.191
*Search frequency*	−0.40	0.27	−0.02	−1.48	0.139

Decreased self-reported *feelings of depression* had a significantly higher association with the *WHO-5 total score (*b = 0.31) than the rest of the variables. Other COVID-related variables with a positive association with *WHO-5 total* score were worrying about a *new lockdown* (b = 0.08) or *losing a loved one* (b = 0.07). On the other hand, worrying about one's *own physical and mental health* (b = −0.11) or *new coronavirus variants* (b = −0.08) and *risk perception: severity* (b = −0.08) were significantly associated with a lower WHO-5 total score. Statistical significance was assured by p < 0.001 for the abovementioned variables; Table 3 includes all the variables with a significant association with the WHO-5 total score. There were no significant differences between WHO-5 total score for non-responders with missing values, when compared with responders (MD = −1.17, p = 0.16).

## 4 Discussion

The main goal of this study was to assess the evolution of self-reported well-being in Spain throughout the second year of the COVID-19 pandemic and beyond, and its association with demographic characteristics and COVID-19 related variables.

The sociodemographic distribution of the participants was similar to Spanish population on January 2022 [43], with a slight underrepresentation of people over 65 years old. Interestingly, despite the deep economic impact of the lockdown in Spain [4], most participants’ economic situation remained stable. Regarding employment status, the study results were according to the national values [44], with an employment rate of 54.4 % in 2022.

There was an increase in Spanish population well-being from May to October 2021, followed by a plateau until late September 2022. The increase in well-being during the initial five-month period accompanied the vaccination campaign in Spain and the decrease in mortality rates. Around 70 % of the population was vaccinated by September 2021 (round 8) and mortality rates were substantially lower than during the first year of the pandemic [8], which may have led people to feel safer, increasing their overall sense of well-being. From October 2021 to February 2022 (round 10), there were no significant differences in reported well-being, in spite of an exponential increase in the number of cases associated with the SARS-CoV-2 Omicron variant during January 2022 [8], possible due to its low lethality rate. Nonetheless, the WHO-5 total scores observed in this study were below the 68-point average observed in the European Quality of Life Survey from 2016 [45]. Although direct comparisons cannot be made, one may hypothesise the reasons why in September 2022 the well-being was still low. At that moment, the “new normality” still included restrictive measures and a certain degree of perceived risk that did not allow for a complete return to pre-pandemic well-being levels [46]. Self-reported compliance with facemask use according to local recommendations, as well as other preventive behaviours related to restrictive measures are reported by round in Supplementary Table 1. One study [18] reported low levels of well-being during the first months of the pandemics in Spain (barely over 50 points), but no other information is available during the first year, as the WHO-5 was not included in the COSMO-Spain study until round 6.

Our study showed increased well-being in the oldest groups, a tendency also seen in Italy [47] and northern Europe [16,29]. Interestingly, before the COVID-19 pandemic, Mediterranean countries showed a decrease in well-being with increased age [45]. Research suggests that greater access to COVID-19 information and media exposure among youth may explain this [47,48]. However, we did not find any association between well-being and COVID-19 information seeking frequency. Other possible explanations may be related to the uncertainty regarding one's future caused by financial self-insufficiency in the youngest groups or the greater impact social isolation may have amongst the youth [16,48]. This generational difference could also result from a higher resilience older people may show due to their life experiences [16,48].

Our results indicated that sex was independently associated with well-being, with women showing significantly lower well-being levels. The relationship between being a woman and experiencing psychological distress during the pandemic has been consistently reported worldwide [48]. This sex disparity is likely multifactorial, although it might relate to a greater incidence of depression or anxiety amongst women [16].

We found education to be a protective factor for well-being, but results throughout the literature are inconsistent [16,48]. A worsened financial situation and being unemployed negatively influenced well-being in our study, in accordance with the literature [16,48]. Possible interventions and support measures developed nationwide should take into consideration these vulnerable groups (women, youth or people with lower socioeconomic status), as they may benefit from targeted action in order to avoid exacerbating the reported disparities.

A significantly high association was found between well-being and feelings of depression in this study: the less depressed a person felt, the higher her or his own well-being. This was foreseeable, as WHO-5 has been validated as a screening tool for mild depression [40,49].

COVID-19 has generated several worries and concerns in the Spanish population [30]. Higher preoccupation with one's physical and mental health was associated with lower well-being, consistent with observations worldwide [50]. Concerns regarding going outside also negatively influenced well-being, which may be explained by the lasting effects of the lockdown [16]. Furthermore, worrying about new coronavirus variants followed the same trend, probably in connection with the appearance of the highly contagious Omicron variant [8]. The impact of these worries on well-being may partially explain why it has not returned to the pre-pandemic levels.

People who were more worried about a new lockdown, the health system overload or losing a loved one showed higher levels of well-being. Concerns about restrictions on freedom of movement may be accompanied by more frequent preventive behaviours, justifying such a positive relation. The same explanation may apply to worries regarding the health system. Further studies are needed to confirm and understand this finding. Finally, the existence of a loved one to care about may explain a greater sense of self-reported wellbeing. Overall, divorcees, widowers and one-person households have been shown to report more mental health problems [16].

Perceived self-efficacy and well-being also went hand-in-hand as shown throughout the pandemic [12,25]. On the contrary, as expected, those with higher risk perception (severity) reported lower levels of well-being [17]. Only hand hygiene and physical distance practices were associated with better well-being levels in this study. The absence of an impact of face mask use on well-being may be explained by a decreasing compliance with its use throughout the pandemic [30,51]. There is ample evidence of the positive influence of good levels of health literacy, and knowledge about health-related topics on well-being and mental health [1,27]. Those who better knew how to follow recommendations and how to proceed in case of a close contact showed higher levels of well-being. This reinforces the need for comprehensive health promotion strategies, with quality concise information during health crises [12,27,28]. Policy makers and administrations, when involving media and digital platforms in health emergencies divulgation strategies, should take this into account [52].

Finally, one should consider the low explained variance of the regression model. The complexity and subjectivity of one's perception of well-being, built in accordance with individual experiences, may partly justify such explained variance, as does the lack of evaluation of past mental health issues.

### 4.1 Strenghts and limitations

There are several strengths and limitations to take into consideration when interpreting this study. The cross-sectional nature of the study does not enable testing for causality, even though the *per round* structure allows analysing the fluctuations of the sense of well-being throughout the pandemic, unlike most one-time cross-sectional studies found in the literature.

The sampling method used in this study also has its pros and cons. The use of a nationwide approach across Spain results in an accurate representation of the public KAPs and well-being, with a relatively large sample, representative of the Spanish population regarding sex, age, education level and large areas of residence. However, the sample size in each round prevented analyses by local regions.

Furthermore, selection bias may arise from the need to access digital technologies to answer the survey, limiting the participation of particularly vulnerable population groups (the oldest old, refugees, migrants or homeless people, for example). People under 18 years old did not participate either. Therefore, one should be careful when extrapolating the results of this study to specific disadvantaged populations. Moreover, the heterogeneity in response rates, with some as low as 36.0 % and others as high as 100.0 %, may also be an important limitation of the study, as it may amplify a selection bias. This should be taken into account especially during vacation periods such as the summer holidays.

Regarding data collection, self-reported behaviours may differ from actual behaviours, even in anonymized surveys, due to people's propensity to answer according to social norms (social desirability bias) [53]. Lastly, not all the scales used in the study have been validated in the context of the COVID-19 pandemic. Regardless, our main goal was measured with the widely used, amply validated WHO-5 scale, which could ease comparisons with other studies using the same instrument.

### 4.2 Conclusions

This study shows an increment in well-being during the second year of the coronavirus pandemic, reaching a plateau from October 2021 onwards, lower than pre-pandemic values. Vulnerable groups at risk of lower well-being levels were younger people, women and people with lower socioeconomic status. They should be prioritized by policymakers in intervention and communication strategies. Self-reported feelings of depression and well-being seem to have an important association. Thus, it is necessary to monitor the mental health status of the population, which might be at risk for a significant increase in pathologies like depression. These can rise with preoccupations regarding one's physical and mental health or other COVID-19 related issues. It can be refrained by empowering people to avoid infection, teaching them how to find accurate information, and knowing what measures and behaviours to follow. Well-being is a complex, highly subjective construct, influenced by a multitude of KAPs, not fully explained by the ones analysed in this study. Further longitudinal studies are necessary in order to evaluate causality and evolution patterns of well-being throughout the COVID-19 pandemic.

## CRediT authorship contribution statement

**Catarina Santos-Ribeiro:** Writing – review & editing, Writing – original draft, Methodology, Investigation, Formal analysis. **Carmen Rodríguez-Blázquez:** Writing – review & editing, Validation, Supervision, Project administration, Methodology, Data curation, Conceptualization. **Alba Ayala:** Writing – review & editing, Methodology, Formal analysis, Data curation. **María Romay-Barja:** Writing – review & editing, Validation, Project administration, Conceptualization. **María Falcón:** Writing – review & editing, Validation, Project administration, Conceptualization. **Maria João Forjaz:** Writing – review & editing, Validation, Resources, Project administration, Investigation, Formal analysis, Conceptualization.

## Data availability

Results from every round are available at the COSMO-Spain website, referenced in the present article (https://portalcne.isciii.es/cosmo-spain/). Further data is available upon request.

## Funding

The COSMO-Spain study was funded by 10.13039/501100004587Carlos III Health Institute. This study was also partially funded by RICAPPS (10.13039/501100004587Carlos III Health Institute, ref: RD21CIII/0003/0002).

## Declaration of competing interest

The authors declare that they have no known competing financial interests or personal relationships that could have appeared to influence the work reported in this paper.

## References

[1] Orfeuvre E., Franck N., Plasse J., Haesebaert F. (2022). Mental well-being in young people with psychiatric disorders during the early phase of COVID-19 lockdown. PLoS One.

[2] (mar 14, 2020). Real Decreto 463/2020, de 14 de marzo, por el que se declara el estado de alarma para la gestión de la situación de crisis sanitaria ocasionada por el COVID-19. https://www.boe.es/buscar/pdf/2020/BOE-A-2020-3692-consolidado.pdf.

[3] Rodríguez-Blázquez C., Falcón M., Romay-Barja M., Ayala A., Forjaz M.J. (2020). Monitorización del comportamiento y las actitudes de la población relacionadas con la COVID-19 en España (COSMO-SPAIN): Estudio OMS [Internet]. COSMO-Spain. https://portalcne.isciii.es/cosmo-spain/.

[4] Pinilla J., Barber P., Vallejo-Torres L., Rodríguez-Mireles S., López-Valcárcel B.G., Serra-Majem L. (28 de abril de 2021). The economic impact of the SARS-COV-2 (COVID-19) pandemic in Spain. Int J Environ Res Public Health.

[5] Centers for Disease Control and Prevention (2021). https://www.cdc.gov/coronavirus/2019-ncov/prevent-getting-sick/prevention-H.pdf.

[6] Gómez Marco J.J., Álvarez Pasquín M.J. (2021). La vacunación COVID-19 en España: aciertos, errores y perspectivas de futuro. Aten Primaria. diciembre de.

[7] Beca-Martínez M.T., Romay-Barja M., Ayala A., Falcon-Romero M., Rodríguez-Blázquez C., Benito A. (noviembre de 2022). Trends in COVID-19 vaccine acceptance in Spain, september 2020‒may 2021. Am J Public Health.

[8] Ritchie H., Mathieu E., Rodés-Guirao L., Appel C., Giattino C., Ortiz-Ospina E. (5 de marzo de 2020). https://ourworldindata.org/coronavirus/country/spain.

[9] Uscher-Pines L., Omer S.B., Barnett D.J., Burke T.A., Balicer R.D. (octubre de 2006). Priority setting for pandemic influenza: an analysis of national preparedness plans. PLoS Med.

[10] World Health Organization (1984). Constitution of the world health organization [internet]. https://apps.who.int/gb/bd/PDF/bd47/EN/constitution-en.pdf?ua=1.

[11] Fusar-Poli P., Salazar de Pablo G., De Micheli A., Nieman D.H., Correll C.U., Kessing L.V. (2020). What is good mental health? A scoping review. Eur Neuropsychopharmacol J Eur Coll Neuropsychopharmacol. febrero de.

[12] World Health Organization (2020).

[13] Blodgett J.M., Birch J.M., Musella M., Harkness F., Kaushal A. (28 de noviembre de 2022). What works to improve wellbeing? A rapid systematic review of 223 interventions evaluated with the warwick-edinburgh mental well-being scales. Int J Environ Res Public Health.

[14] Bhatia N., Heim J., Schenkel B., Vasquez J.G. (2023). Quality of life and patient-reported symptoms in a Phase 4, real-world study of tildrakizumab in patients with moderate-to-severe psoriasis: week 28 interim analysis. J Dermatol Treat. 31 de diciembre de.

[15] Topp C.W., Østergaard S.D., Søndergaard S., Bech P. (2015). The WHO-5 well-being index: a systematic review of the literature. Psychother Psychosom.

[16] Tsai F.Y., Schillok H., Coenen M., Merkel C., Jung-Sievers C. (9 de marzo de 2022). The well-being of the German adult population measured with the WHO-5 over different phases of the COVID-19 pandemic: an analysis within the COVID-19 Snapshot monitoring study (COSMO). Int J Environ Res Public Health.

[17] de Girolamo G., Ferrari C., Candini V., Buizza C., Calamandrei G., Caserotti M. (2022). Psychological well-being during the COVID-19 pandemic in Italy assessed in a four-waves survey. Sci Rep. 26 de octubre de.

[18] Wilke J., Hollander K., Mohr L., Edouard P., Fossati C., González-Gross M. (2021). Drastic reductions in mental well-being observed globally during the COVID-19 pandemic: results from the ASAP survey. Front Med.

[19] Romay-Barja M., Pascual-Carrasco M., De Tena-Dávila M.J., Falcón M., Rodriguez-Blazquez C., Forjaz M.J. (20 de mayo de 2021). How patients with COVID-19 managed the disease at home during the first wave in Spain: a cross-sectional study. BMJ Open.

[20] Pierce M., Hope H., Ford T., Hatch S., Hotopf M., John A. (2020). Mental health before and during the COVID-19 pandemic: a longitudinal probability sample survey of the UK population. Lancet Psychiatry. octubre de.

[21] Chigwedere O.C., Sadath A., Kabir Z., Arensman E. (22 de junio de 2021). The impact of epidemics and pandemics on the mental health of healthcare workers: a systematic review. Int J Environ Res Public Health.

[22] Al Maqbali M., Al Sinani M., Al-Lenjawi B. (2021). Prevalence of stress, depression, anxiety and sleep disturbance among nurses during the COVID-19 pandemic: a systematic review and meta-analysis. J Psychosom Res.

[23] Vindegaard N., Benros M.E. (2020). COVID-19 pandemic and mental health consequences: systematic review of the current evidence. Brain Behav Immun. octubre de.

[24] Michie S., West R., Harvey N. (2 de noviembre de 2020). The concept of “fatigue” in tackling covid-19. BMJ.

[25] Forjaz M.J., Romay Barja M., Falcón Romero M., Rodriguez-Blazquez C. (2021). Spain COVID-19 Snapshot MOnitoring (COSMO Spain): monitoring knowledge, risk perceptions, preventive behaviours, and public trust in the current coronavirus outbreak in Spain. Psycharchives.

[26] Büssing A., Rodrigues Recchia D., Hein R., Dienberg T. (2020). Perceived changes of specific attitudes, perceptions and behaviors during the Corona pandemic and their relation to wellbeing. Health Qual Life Outcomes.

[27] Elbarazi I., Saddik B., Grivna M., Aziz F., Elsori D., Stip E. (22 de febrero de 2022). The impact of the COVID-19 “infodemic” on well-being: a cross-sectional study. J Multidiscip Healthc.

[28] Zarocostas J. (2020). How to fight an infodemic. The Lancet. 29 de febrero de.

[29] Every-Palmer S., Jenkins M., Gendall P., Hoek J., Beaglehole B., Bell C. (2020). Psychological distress, anxiety, family violence, suicidality, and wellbeing in New Zealand during the COVID-19 lockdown: a cross-sectional study. PloS One.

[30] Rodríguez-Blázquez C., Romay-Barja M., Falcón M., Ayala A., Forjaz M.J. (31 de mayo de 2021). The COSMO-Spain survey: three first rounds of the WHO behavioral insights tool. Front Public Health.

[31] INE (23 de julio de 2024). INEbase/Demografía y población/Cifras de población y Censos demográficos/Estadística continua de población/Últimos datos. https://www.ine.es/dyngs/INEbase/es/operacion.htm?c=Estadistica_C&cid=1254736177095&menu=ultiDatos&idp=1254735572981.

[32] Centro de Coordinación de Alertas y Emergencias Sanitarias (2021). Actualización n^o^ 370 07.05.2021 [Internet].

[33] Centro de Coordinación de Alertas y Emergencias Sanitarias (2021). Actualización n^o^ 434 05.08.2021 [Internet].

[34] Centro de Coordinación de Alertas y Emergencias Sanitarias (2021). Actualización n^o^ 476. 04.10.2021 [Internet].

[35] Centro de Coordinación de Alertas y Emergencias Sanitarias (2021). Actualización n^o^ 520 10.12.2021 [Internet].

[36] Centro de Coordinación de Alertas y Emergencias Sanitarias (2022). Actualización n^o^ 572 25.02.2022 [Internet].

[37] Actualizacion_594_COVID-19.pdf [Internet]. [citado 27 de enero de 2023]. Disponible en: https://www.sanidad.gob.es/profesionales/saludPublica/ccayes/alertasActual/nCov/documentos/Actualizacion_594_COVID-19.pdf.

[38] Actualizacion_638_COVID-19.pdf [Internet]. [citado 13 de diciembre de 2022]. Disponible en: https://www.sanidad.gob.es/profesionales/saludPublica/ccayes/alertasActual/nCov/documentos/Actualizacion_638_COVID-19.pdf.

[39] Bech P. (1999). Health-related quality of life measurements in the assessment of pain clinic results. Acta Anaesthesiol Scand.

[40] Lucas-Carrasco R., Allerup P., Bech P. (2012). The validity of the WHO-5 as an early screening for apathy in an elderly population. Curr Gerontol Geriatr Res.

[41] Rodríguez-Blázquez C., Romay-Barja M., Falcón M., Ayala A., Forjaz M. (8 Sep. 2022). Psychometric properties of the COVID-19 Pandemic Fatigue Scale: results from the online COSMO-Spain survey. JMIR Public Health Surveill..

[42] Falcón M., Rodríguez-Blázquez C., Fernández-Gutiérrez M., Romay-Barja M., Bas-Sarmiento P., Forjaz M.J. (27 de septiembre de 2022). Measuring COVID-19 health literacy: validation of the COVID-19 HL questionnaire in Spain. Health Qual Life Outcomes.

[43] Instituto Nacional de Estadística (2022). Población residente por fecha, sexo y edad [Internet].

[44] Instituto Nacional de Estadística (2022). Tasas de actividad, paro y empleo por provincia y sexo. https://ine.es/jaxiT3/Datos.htm?t=3996.

[45] Jungblut J.M., Anderson R. (2019).

[46] Gruber J., Clark L.A., Abramowitz J.S., Aldao A., Chung T., Forbes E.E. (2021). Mental health and clinical psychological science in the time of COVID-19: challenges, opportunities, and a call to action. Am Psychol. abril de.

[47] Mazza C., Ricci E., Biondi S., Colasanti M., Ferracuti S., Napoli C. (enero de 2020). A nationwide survey of psychological distress among Italian people during the COVID-19 pandemic: immediate psychological responses and associated factors. Int J Environ Res Public Health.

[48] Wang Y., Kala M.P., Jafar T.H. (2020). Factors associated with psychological distress during the coronavirus disease 2019 (COVID-19) pandemic on the predominantly general population: a systematic review and meta-analysis. PLOS ONE. dic de.

[49] Lucas-Carrasco R. (2012). Reliability and validity of the Spanish version of the world health organization-five well-being index in elderly. Psychiatry Clin Neurosci.

[50] Meltzer G.Y., Chang V.W., Lieff S.A., Grivel M.M., Yang L.H., Des Jarlais D.C. (30 de octubre de 2021). Behavioral correlates of COVID-19 worry: stigma, knowledge, and news source. Int J Environ Res Public Health.

[51] Wright L., Fancourt D. (2021). Do predictors of adherence to pandemic guidelines change over time? A panel study of 22,000 UK adults during the COVID-19 pandemic. Prev Med. diciembre de.

[52] Larson H.J. (2018). The biggest pandemic risk? Viral misinformation. Nature. octubre de.

[53] Tourangeau R., Yan T. (2007). Sensitive questions in surveys. Psychol Bull.

