# Supplementary material for: Evolution of Spanish population well-being during the COVID-19 pandemic: Results from the COSMO-Spain study
Source: Heliyon. 2025 Jan 31;11(3):e42409. doi: 10.1016/j.heliyon.2025.e42409 (PMC11847275; doi:10.1016/j.heliyon.2025.e42409)
Supplement: Multimedia component 1 [file mmc1.docx]

| **Supplementary table 1:** Variables included in the present study. | | | |
| --- | --- | --- | --- |
|  |  | |  |
| **Variable group** | **Variable** | | **Answering options** |
| **Dependent variable** | | | |
| **WHO-5** | *WHO-5 total score* | | Scale from 0 (worst possible wellbeing) to 100 (best possible wellbeing) |
| **Independent variables** | | | |
| **Demographics** | *Sex* | | Female |
|  |  |  | Male |
|  | *Age group (years old)* | | 18 to 24 |
|  |  |  | 25 to 34 |
|  |  |  | 35 to 49 |
|  |  |  | 50 to 64 |
|  |  |  | 65+ |
|  | *Education level* | | Incomplete primary or less |
|  |  |  | Primary |
|  |  |  | Secondary |
|  |  |  | University |
|  | *Economic situation (during the previous three months)* | | Has improved |
|  |  |  | Remains the same |
|  |  |  | Has worsened |
|  | *Employment status* | | Working a high to moderate risk of contagion job |
|  |  |  | Working a low risk of contagion job |
|  |  |  | Student |
|  |  |  | Retired/pensioner/homemaker |
|  |  |  | Unemployed/ERTE |
| **Worrying** | *Concern about COVID-19* | | Scale from 1 (not concerned at all) to 5 (extremely concerned) |
|  | *Speed of propagation* | | Scale from 1 (spreading slowly) to 5 (spreading fast) |
|  | *Feelings of depression* | | Scale from 1 (makes me feel depressed) to 5 (does not affect my mood) |
|  | *Feelings of fear* | | Scale from 1 (makes me feel fear) to 5 (does not make me feel fear at all) |
|  | *Health system overload* | | Scale from 1 (not worried at all) to 5 (worried a lot) |
|  | *Own physical and mental health* | |  |
|  | *Going outside* | |  |
|  | *People that do not wear face masks* | |  |
|  | *A new lockdown* | |  |
|  | *Losing a loved one* | |  |
|  | *Becoming unemployed* | |  |
|  | *New coronavirus variants* | |  |
| **Risk perception** | *Severity* | | Scale from 1 (very light) to 5 (very severe) |
|  | *Probability* | | Scale from 1 (highly unlikely) to 5 (highly likely) |
|  | *Self-efficacy at avoiding COVID-19* | | Scale from 1 (very hard) to 5 (very easy) |
| **Preventive behaviors** | *Frequent hand hygiene* | | Scale from 1 (never) to 5 (always) |
|  | *Hydroalcoholic gel use* | |  |
|  | *Physical distancing* | |  |
|  | *Using masks* | |  |
| **Pandemic fatigue (CPFS)** | *Pandemic fatigue* | | Final score ranging from 6 (lowest possible level) to 30 (highest possible level of pandemic fatigue) |
| **Health literacy** | *Following recommendations about protecting oneself* | | Scale from 1 (very hard) to 4 (very easy) |
|  | *Valuing if media information is reliable* | |  |
|  | *Understanding what to do when one is a close contact* | |  |
|  | *Finding information regarding vaccines* | |  |
|  | *Understanding risks and benefits of vaccination* | |  |
|  | *Assessing if media information regarding vaccines is reliable* | |  |
|  | *Deciding if one should get vaccinated* | |  |
| **Information search behavior** | *Information search frequency* | | Scale from 1 (never) to 5 (several times a day) |
|  | *Trust in sources of information* | *TV News* | Scale from 1 (very little trust) to 5 (strong sense of trust) |
|  |  | *Debate programs* |  |
|  |  | *Press conferences* |  |
|  |  | *National press* |  |
|  |  | *Healthcare professionals* |  |
|  |  | *Social media* |  |
|  |  | *Internet* |  |
|  |  | *Health Ministry* |  |
|  |  | *WHO* |  |
|  |  | *Help phone lines* |  |
|  |  | *Radio* |  |
| **Trust in institutions** | *Primary care centers* | | Scale from 1 (very little trust) to 5 (strong sense of trust) |
|  | *Workplace* | |  |
|  | *Hospitals* | |  |
|  | *Health Ministry* | |  |
|  | *Regional Government* | |  |
|  | *Scientists* | |  |
|  | *Education centers* | |  |
|  | *Public transport* | |  |
|  | *Press* | |  |
|  | *Central Government* | |  |

| **Supplementary table 2:** round-specific independent variables descriptive statistics. SD: standard deviation | | | | | | | | | | | | |  |  |  |  |  |  |  |  |  |  |
| --- | --- | --- | --- | --- | --- | --- | --- | --- | --- | --- | --- | --- | --- | --- | --- | --- | --- | --- | --- | --- | --- | --- |
|  |  |  |  |  |  |  |  |  |  |  |  |  |  |  |  |  |  |  |  |  |  |  |
| ***Variable*** | Categories |  | **Round 6 (n=1,001)** | |  | **Round 7 (n=1,000)** | |  | **Round 8 (n=1,042)** | |  | **Round 9 (n=1,049)** | |  | **Round 10 (n=1,067)** | |  | **Round 11 (n=1,056)** | |  | **Round 12 (n=1,051)** | |
|  |  |  | **n** | **%** |  | **n** | **%** |  | **n** | **%** |  | **n** | **%** |  | **n** | **%** |  | **n** | **%** |  | **n** | **%** |
| ***Sex*** | Female |  | 501 | 50.0% |  | 500 | 50.0% |  | 521 | 50.0% |  | 525 | 50.0% |  | 534 | 50.0% |  | 528 | 50.0% |  | 525 | 50.0% |
|  | Male |  | 500 | 50.0% |  | 500 | 50.0% |  | 521 | 50.0% |  | 524 | 50.0% |  | 533 | 50.0% |  | 528 | 50.0% |  | 526 | 50.0% |
| ***Age group*** *(years old)* | 18 to 24 |  | 63 | 6.3% |  | 73 | 7.3% |  | 73 | 7.0% |  | 10 | 1.0% |  | 107 | 10.1% |  | 91 | 8.6% |  | 80 | 7.6% |
|  | 25 to 34 |  | 172 | 17.2% |  | 144 | 14.4% |  | 167 | 16.0% |  | 226 | 21.5% |  | 167 | 15.6% |  | 188 | 17.8% |  | 187 | 17.8% |
|  | 35 to 49 |  | 347 | 34.6% |  | 377 | 37.7% |  | 365 | 35.0% |  | 381 | 36.3% |  | 309 | 29.0% |  | 321 | 30.4% |  | 303 | 28.9% |
|  | 50 to 64 |  | 304 | 30.4% |  | 301 | 30.1% |  | 305 | 29.3% |  | 299 | 28.5% |  | 350 | 32.8% |  | 328 | 31.1% |  | 356 | 33.9% |
|  | 65+ |  | 115 | 11.5% |  | 105 | 10.5% |  | 133 | 12.8% |  | 133 | 12.7% |  | 133 | 12.5% |  | 127 | 12.0% |  | 124 | 11.8% |
| ***Education level*** | Incomplete primary or less |  | 171 | 17.1% |  | 173 | 17.3% |  | 180 | 17.3% |  | 181 | 17.3% |  | 179 | 16.8% |  | 177 | 16.8% |  | 177 | 16.8% |
|  | Primary |  | 285 | 28.5% |  | 279 | 27.9% |  | 291 | 27.9% |  | 293 | 27.9% |  | 278 | 26.1% |  | 276 | 26.1% |  | 274 | 26.1% |
|  | Secondary |  | 227 | 22.7% |  | 230 | 23.0% |  | 240 | 23.0% |  | 241 | 23.0% |  | 264 | 24.7% |  | 261 | 24.7% |  | 260 | 24.7% |
|  | University |  | 317 | 31.7% |  | 318 | 31.8% |  | 331 | 31.8% |  | 334 | 31.8% |  | 346 | 32.4% |  | 342 | 32.4% |  | 341 | 32.4% |
| ***Economic situation*** (during the previous three months) | Has improved |  | 88 | 8.8% |  | 85 | 8.5% |  | 93 | 8.9% |  | 88 | 8.4% |  | 107 | 10.1% |  | 100 | 9.5% |  | 139 | 13.2% |
|  | Remains the same |  | 678 | 67.7% |  | 713 | 71.3% |  | 716 | 68.7% |  | 700 | 66.7% |  | 680 | 63.7% |  | 641 | 60.7% |  | 584 | 55.6% |
|  | Has worsened |  | 236 | 23.5% |  | 202 | 20.2% |  | 233 | 22.4% |  | 261 | 24.8% |  | 280 | 26.3% |  | 315 | 29.8% |  | 328 | 31.2% |
| ***Employment status*** | Working a high to moderate risk of contagion job |  | 382 | 38.1% |  | 414 | 41.4% |  | 401 | 38.5% |  | 473 | 45.1% |  | 358 | 33.6% |  | 351 | 33.3% |  | 378 | 36.0% |
|  | Working a low risk of contagion job |  | 142 | 14.2% |  | 161 | 16.1% |  | 165 | 15.8% |  | 166 | 15.8% |  | 177 | 16.6% |  | 180 | 17.1% |  | 199 | 18.9% |
|  | Student |  | 63 | 6.3% |  | 52 | 5.2% |  | 58 | 5.5% |  | 39 | 3.7% |  | 86 | 8.0% |  | 95 | 9.0% |  | 81 | 7.7% |
|  | Retired/pensioner/homemaker |  | 261 | 26.1% |  | 247 | 24.7% |  | 270 | 25.9% |  | 249 | 23.7% |  | 266 | 24.9% |  | 275 | 26.0% |  | 254 | 24.2% |
|  | Unemployed/ERTE |  | 153 | 15.3% |  | 127 | 12.7% |  | 148 | 14.2% |  | 121 | 11.5% |  | 180 | 16.9% |  | 155 | 14.7% |  | 139 | 13.2% |
|  |  |  | **Mean** | **SD** |  | **Mean** | **SD** |  | **Mean** | **SD** |  | **Mean** | **SD** |  | **Mean** | **SD** |  | **Mean** | **SD** |  | **Mean** | **SD** |
| ***WHO-5 total score*** (0-100) | |  | 51.52 | 21.94 |  | 53.15 | 22.13 |  | 55.25 | 22.23 |  | 55.14 | 22.42 |  | 54.10 | 23.13 |  | 56.29 | 23.79 |  | 55.76 | 23.60 |
| ***Concern about COVID-19***(1-5) | |  | 3.43 | 1.09 |  | 3.53 | 1.05 |  | 3.24 | 1.13 |  | 3.38 | 1.08 |  | 2.97 | 1.15 |  | 2.72 | 1.10 |  | 2.50 | 1.07 |
| ***Speed of propagation*** (1-5) | |  | 3.40 | 1.18 |  | 4.17 | 0.97 |  | 2.95 | 1.14 |  | 3.78 | 1.09 |  | 3.66 | 1.16 |  | 3.40 | 1.17 |  | 2.73 | 1.14 |
| ***Feelings of depression*** (1-5) | |  | 2.91 | 1.24 |  | 2.86 | 1.19 |  | 3.07 | 1.22 |  | 3.05 | 1.22 |  | 3.08 | 1.28 |  | 3.23 | 1.29 |  | 3.30 | 1.25 |
| ***Feelings of fear*** (1-5) | |  | 2.84 | 1.25 |  | 2.80 | 1.22 |  | 2.98 | 1.21 |  | 2.90 | 1.22 |  | 3.09 | 1.31 |  | 3.19 | 1.22 |  | 3.26 | 1.19 |
| **Worry** (1-5) | ***Health system overload*** |  | 4.34 | 0.97 |  | 4.38 | 0.94 |  | 4.17 | 1.09 |  | 4.34 | 0.95 |  | 4.14 | 1.11 |  | 4.01 | 1.12 |  | 3.98 | 1.17 |
|  | ***Own physical and mental health*** |  | 3.83 | 1.27 |  | 3.89 | 1.21 |  | 3.71 | 1.29 |  | 3.77 | 1.26 |  | 3.80 | 1.30 |  | 3.67 | 1.30 |  | 3.45 | 1.40 |
|  | ***Going outside*** |  | 2.85 | 1.33 |  | 2.82 | 1.29 |  | 2.59 | 1.30 |  | 2.70 | 1.32 |  | 2.51 | 1.31 |  | 2.33 | 1.32 |  | 2.06 | 1.22 |
|  | ***A new lockdown*** |  | 3.82 | 1.35 |  | 3.79 | 1.33 |  | 3.70 | 1.37 |  | 3.80 | 1.30 |  | 3.59 | 1.44 |  | 3.63 | 1.40 |  | 3.44 | 1.49 |
|  | ***Losing a loved one*** |  | 4.58 | 0.90 |  | 4.53 | 0.94 |  | 4.48 | 1.01 |  | 4.54 | 0.89 |  | 4.36 | 1.07 |  | 4.35 | 1.06 |  | 4.25 | 1.16 |
|  | ***Becoming unemployed*** |  | 3.16 | 1.63 |  | 3.22 | 1.63 |  | 3.12 | 1.60 |  | 3.07 | 1.63 |  | 3.10 | 1.65 |  | 2.98 | 1.63 |  | 2.83 | 1.62 |
|  | ***New coronavirus variants*** |  | 4.12 | 1.05 |  | 4.19 | 1.07 |  | 3.90 | 1.15 |  | 3.99 | 1.12 |  | 3.79 | 1.22 |  | 3.68 | 1.22 |  | 3.38 | 1.24 |
| ***Risk perception: severity*** (1-5) | |  | 3.11 | 0.98 |  | 2.99 | 0.94 |  | 2.97 | 0.95 |  | 2.95 | 0.94 |  | 2.78 | 0.97 |  | 2.71 | 0.91 |  | 2.59 | 0.89 |
| ***Risk perception: probability*** (1-5) | |  | 2.60 | 1.09 |  | 2.71 | 1.08 |  | 2.63 | 1.08 |  | 2.83 | 1.10 |  | 3.08 | 1.14 |  | 3.09 | 1.12 |  | 2.96 | 1.10 |
| ***Self-efficacy at avoiding COVID-19*** (1-5) | |  | 3.26 | 0.96 |  | 3.09 | 0.96 |  | 3.23 | 0.96 |  | 3.07 | 0.98 |  | 3.17 | 1.05 |  | 3.19 | 1.06 |  | 3.25 | 1.04 |
| ***Frequent hand hygiene*** (1-5) | |  | 4.23 | 1.04 |  | 4.20 | 1.03 |  | 4.10 | 1.11 |  | 4.04 | 1.11 |  | 3.90 | 1.24 |  | 3.85 | 1.22 |  | 3.65 | 1.32 |
| ***Hydroalcoholic gel use*** (1-5) | |  | 4.41 | 0.96 |  | 4.27 | 1.01 |  | 4.16 | 1.12 |  | 4.08 | 1.12 |  | 4.02 | 1.21 |  | 3.66 | 1.32 |  | 3.34 | 1.43 |
| ***Physical distancing*** (1-5) | |  | 4.03 | 1.03 |  | 4.09 | 0.96 |  | 3.78 | 1.10 |  | 3.77 | 1.12 |  | 3.69 | 1.22 |  | 3.38 | 1.25 |  | 2.95 | 1.32 |
| ***Using masks*** (1-5) | |  | 4.71 | 0.71 |  | 4.64 | 0.72 |  | 4.48 | 0.92 |  | 4.53 | 0.86 |  | 4.46 | 0.97 |  | 4.44 | 1.05 |  | 4.33 | 1.08 |
| ***Pandemic fatigue*** (6-30) | |  | 17.47 | 5.11 |  | 17.60 | 5.41 |  | 17.33 | 5.27 |  | 17.47 | 5.41 |  | 18.52 | 5.52 |  | 18.07 | 5.61 |  | 17.48 | 5.59 |
| **Health literacy** (1-4) | ***Following recommendations about protecting oneself*** |  | 3.31 | 0.71 |  | 3.28 | 0.68 |  | 3.30 | 0.72 |  | 3.28 | 0.74 |  | 3.27 | 0.70 |  | 3.29 | 0.69 |  | 3.28 | 0.75 |
|  |  |  | (n=975) | |  | (n=978) | |  | (n=1,020) | |  | (n=1,027) | |  | (n=1,045) | |  | (n=1,021) | |  | (n=1,023) | |
|  | ***Valuing if media information is reliable*** |  | 2.44 | 0.94 |  | 2.52 | 0.93 |  | 2.58 | 0.92 |  | 2.57 | 0.95 |  | 2.62 | 0.94 |  | 2.66 | 0.93 |  | 2.65 | 0.96 |
|  |  |  | (n=927) | |  | (n=918) | |  | (n=981) | |  | (n=974) | |  | (n=988) | |  | (n=990) | |  | (n=994) | |
|  | ***Understanding what to do when one is a close contact*** |  | 3.21 | 0.72 |  | 3.19 | 0.76 |  | 3.14 | 0.78 |  | 3.18 | 0.76 |  | 3.12 | 0.76 |  | 3.17 | 0.78 |  | 3.21 | 0.75 |
|  |  |  | (n=968) | |  | (n=971) | |  | (n=1,007) | |  | (n=1,017) | |  | (n=1,040) | |  | (n=1,017) | |  | (n=1,017) | |
|  | ***Finding information regarding vaccines*** |  | 2.77 | 0.88 |  | 2.95 | 0.84 |  | 3.01 | 0.83 |  | 2.99 | 0.86 |  | 2.98 | 0.87 |  | 3.01 | 0.88 |  | 2.97 | 0.88 |
|  |  |  | (n=946) | |  | (n=946) | |  | (n=990) | |  | (n=994) | |  | (n=1,012) | |  | (n=999) | |  | (n=1,009) | |
|  | ***Understanding risks and benefits of vaccination*** |  | 3.12 | 0.88 |  | 3.21 | 0.86 |  | 3.26 | 0.84 |  | 3.31 | 0.79 |  | 3.2 | 0.86 |  | 3.20 | 0.86 |  | 3.13 | 0.87 |
|  |  |  | (n=961) | |  | (n=957) | |  | (n=1,004) | |  | (n=1,013) | |  | (n=1,027) | |  | (n=1,007) | |  | (n=1,010) | |
|  | ***Assessing if media information regarding vaccines is reliable*** |  | 2.45 | 0.94 |  | 2.59 | 0.96 |  | 2.65 | 0.92 |  | 2.63 | 0.95 |  | 2.67 | 0.94 |  | 2.71 | 0.96 |  | 2.63 | 0.92 |
|  |  |  | (n=930) | |  | (n=921) | |  | (n=972) | |  | (n=967) | |  | (n=999) | |  | (n=979) | |  | (n=987) | |
|  | ***Deciding if one should get vaccinated*** |  | 3.33 | 0.89 |  | 3.40 | 0.84 |  | 3.44 | 0.81 |  | 3.49 | 0.78 |  | 3.37 | 0.84 |  | 3.34 | 0.83 |  | 3.26 | 0.87 |
|  |  |  | (n=956) | |  | (n=970) | |  | (n=1,012) | |  | (n=1,021) | |  | (n=1,035) | |  | (n=1,014) | |  | (n=1,007) | |
| ***Information search frequency*** (1-5) | |  | 2.85 | 1.08 |  | 3.02 | 1.10 |  | 2.72 | 1.02 |  | 2.80 | 1.03 |  | 2.77 | 1.11 |  | 2.59 | 1.06 |  | 2.30 | 1.04 |
| **Trust in sources of information (1-5)** | ***TV News*** |  | 2.70 | 1.15 |  | 2.77 | 1.19 |  | 2.77 | 1.16 |  | 2.79 | 1.18 |  | 2.87 | 1.21 |  | 2.76 | 1.17 |  | 2.66 | 1.16 |
|  | ***Debate programs*** |  | 2.46 | 1.11 |  | 2.44 | 1.14 |  | 2.46 | 1.08 |  | 2.48 | 1.15 |  | 2.65 | 1.13 |  | 2.52 | 1.13 |  | 2.48 | 1.12 |
|  | ***Press conferences*** |  | 2.64 | 1.15 |  | 2.63 | 1.15 |  | 2.72 | 1.17 |  | 2.66 | 1.17 |  | 2.76 | 1.14 |  | 2.68 | 1.10 |  | 2.63 | 1.15 |
|  | ***National press*** |  | 2.63 | 1.10 |  | 2.67 | 1.10 |  | 2.69 | 1.09 |  | 2.66 | 1.09 |  | 2.77 | 1.11 |  | 2.68 | 1.12 |  | 2.61 | 1.10 |
|  | ***Healthcare professionals*** |  | 3.86 | 1.08 |  | 3.86 | 1.13 |  | 3.88 | 1.12 |  | 3.79 | 1.15 |  | 3.94 | 1.12 |  | 3.89 | 1.08 |  | 3.86 | 1.11 |
|  | ***Social media*** |  | 2.01 | 1.04 |  | 1.98 | 1.04 |  | 2.02 | 1.05 |  | 1.98 | 1.05 |  | 2.19 | 1.11 |  | 2.12 | 1.05 |  | 2.07 | 1.04 |
|  | ***Internet*** |  | 2.38 | 1.03 |  | 2.35 | 1.04 |  | 2.43 | 1.05 |  | 2.36 | 1.08 |  | 2.48 | 1.07 |  | 2.49 | 1.07 |  | 2.41 | 1.08 |
|  | ***Health Ministry*** |  | 3.08 | 1.28 |  | 3.23 | 1.27 |  | 3.23 | 1.28 |  | 3.25 | 1.28 |  | 3.28 | 1.29 |  | 3.24 | 1.25 |  | 3.22 | 1.27 |
|  | ***WHO*** |  | 3.16 | 1.21 |  | 3.19 | 1.24 |  | 3.25 | 1.24 |  | 3.22 | 1.23 |  | 3.33 | 1.25 |  | 3.31 | 1.25 |  | 3.30 | 1.27 |
|  | ***Help phone lines*** |  | 2.96 | 1.17 |  | 3.03 | 1.21 |  | 3.11 | 1.20 |  | 3.04 | 1.22 |  | 3.15 | 1.23 |  | 3.12 | 1.18 |  | 3.08 | 1.18 |
|  | ***Radio*** |  | 2.76 | 1.08 |  | 2.76 | 1.11 |  | 2.80 | 1.09 |  | 2.78 | 1.11 |  | 2.87 | 1.11 |  | 2.78 | 1.10 |  | 2.68 | 1.11 |
| **Trust in institutions (1-5)** | ***Primary care centers*** |  | 3.41 | 1.10 |  | 3.38 | 1.14 |  | 3.40 | 1.14 |  | 3.39 | 1.14 |  | 3.49 | 1.14 |  | 3.50 | 1.13 |  | 3.46 | 1.15 |
|  | ***Workplace*** |  | 3.07 | 1.15 |  | 3.06 | 1.17 |  | 3.08 | 1.14 |  | 3.06 | 1.16 |  | 3.10 | 1.17 |  | 3.05 | 1.13 |  | 3.01 | 1.15 |
|  | ***Hospitals*** |  | 3.60 | 1.08 |  | 3.58 | 1.11 |  | 3.58 | 1.12 |  | 3.53 | 1.12 |  | 3.66 | 1.10 |  | 3.63 | 1.09 |  | 3.59 | 1.15 |
|  | ***Health Ministry*** |  | 2.94 | 1.23 |  | 3.01 | 1.25 |  | 3.08 | 1.25 |  | 3.07 | 1.25 |  | 3.15 | 1.26 |  | 3.08 | 1.28 |  | 3.10 | 1.27 |
|  | ***Regional Government*** |  | 3.05 | 1.14 |  | 2.93 | 1.14 |  | 3.01 | 1.17 |  | 2.92 | 1.16 |  | 3.04 | 1.19 |  | 2.99 | 1.15 |  | 2.91 | 1.18 |
|  | ***Scientists*** |  | 3.86 | 1.08 |  | 3.81 | 1.14 |  | 3.80 | 1.15 |  | 3.81 | 1.14 |  | 3.92 | 1.10 |  | 3.80 | 1.10 |  | 3.81 | 1.13 |
|  | ***Education centers*** |  | 3.16 | 1.03 |  | 3.11 | 1.10 |  | 3.15 | 1.05 |  | 3.02 | 1.05 |  | 3.14 | 1.10 |  | 3.12 | 1.05 |  | 3.02 | 1.05 |
|  | ***Public transport*** |  | 2.18 | 1.02 |  | 2.26 | 1.07 |  | 2.33 | 1.07 |  | 2.21 | 1.05 |  | 2.47 | 1.14 |  | 2.57 | 1.13 |  | 2.69 | 1.11 |
|  | ***Press*** |  | 2.42 | 1.04 |  | 2.43 | 1.04 |  | 2.41 | 1.06 |  | 2.41 | 1.05 |  | 2.52 | 1.09 |  | 2.46 | 1.07 |  | 2.40 | 1.09 |
|  | ***Central Government*** |  | 2.42 | 1.20 |  | 2.48 | 1.23 |  | 2.49 | 1.26 |  | 2.57 | 1.27 |  | 2.60 | 1.28 |  | 2.60 | 1.24 |  | 2.60 | 1.28 |
